# PUF-8, a Pumilio Homolog, Inhibits the Proliferative Fate in the *Caenorhabditis elegans* Germline

**DOI:** 10.1534/g3.112.003350

**Published:** 2012-10-01

**Authors:** Hilary Racher, Dave Hansen

**Affiliations:** Department of Biological Sciences, University of Calgary, Calgary, Alberta, Canada, T2N 1N4

**Keywords:** *puf-8*, Pumilio, PUF, germline stem cells, proliferative fate, Notch signaling

## Abstract

Stem cell populations are maintained by keeping a balance between self-renewal (proliferation) and differentiation of dividing stem cells. Within the *Caenorhabditis elegans* germline, the key regulator maintaining this balance is the canonical Notch signaling pathway, with GLP-1/Notch activity promoting the proliferative fate. We identified the Pumilio homolog, PUF-8, as an inhibitor of the proliferative fate of stem cells in the *C. elegans* germline. *puf-8(0)* strongly enhances overproliferation of *glp-1(gf)* mutants and partially suppresses underproliferation of a weak *glp-1(lf)* mutant. The germline tumor that is formed in a *puf-8(0)*; *glp-1(gf)* double mutant is due to a failure of germ cells to enter meiotic prophase. *puf-8* likely inhibits the proliferative fate through negatively regulating GLP-1/Notch signaling or by functioning parallel to it.

Successful reproduction in many organisms hinges on their ability to produce gametes throughout much of their adult lives. Prolonged gamete production relies on the maintenance of a population of undifferentiated germline stem cells (GSCs). A balance between proliferation (self-renewal) and differentiation (meiotic entry) allows GSCs to be maintained while still producing the required gametes. The *C. elegans* germline has emerged as a powerful model to study the control of this balance. Located at the distal end of each hermaphrodite gonad arm is a population of ~200 to 250 mitotically dividing cells, with approximately 30% to 40% of these cells being in premeiotic S-phase (Fox *et al.* 2011; Killian and Hubbard 2005; Lamont *et al.* 2004). The proliferative fate of the stem cells is maintained in the distal gonad primarily through the activity of the conserved GLP-1/Notch signaling pathway (Figure 1) (Hansen and Schedl 2006, 2012; Kimble and Crittenden 2007). GLP-1/Notch signaling likely activates the transcription of genes required to promote the proliferative fate, which genes are yet to be fully described. GLP-1/Notch signaling levels are thought to decrease as cells move proximally, allowing the activities of at least two downstream genetic pathways to increase (Eckmann *et al.* 2004; Hansen *et al.* 2004b; Kadyk and Kimble 1998). These two pathways, the *gld-1* and *gld-2* pathways, promote entry into meiosis and/or inhibit the proliferative fate (Figure 1), and function redundantly; when one pathway is inactivated, the other pathway is sufficient to promote entry into meiosis and/or inhibit the proliferative fate. However, if both pathways are inactivated, little or no entry into meiosis occurs, and a germline tumor of proliferative germ cells is formed (Eckmann *et al.* 2004; Hansen *et al.* 2004b; Kadyk and Kimble 1998). Inhibition of the *gld-1* and *gld-2* pathways in the distal end of the gonad is accomplished, at least in part, through the activities of *fbf-1* and *fbf-2*, which encode homologs of Drosophila Pumilio. FBF inhibits these pathways, at least in part, through binding to the 3′UTRs of *gld-1* and *gld-3*, preventing their translation (Eckmann *et al.* 2004; Suh *et al.* 2009)

**Figure 1:**
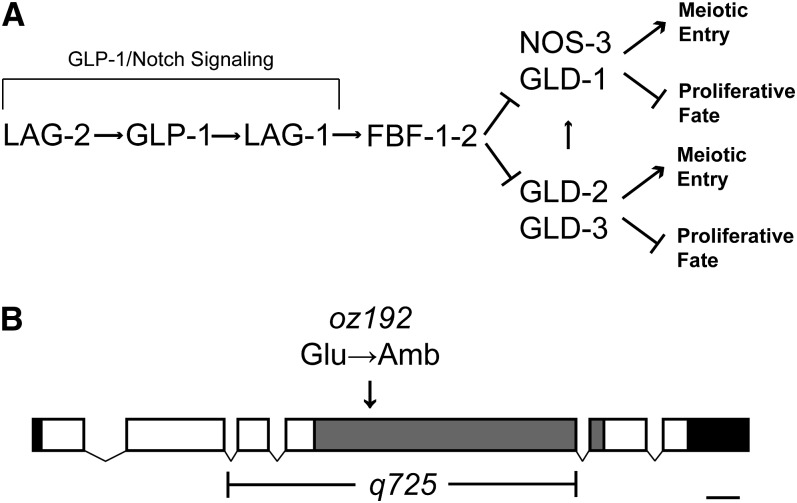
Proliferation *vs.* meiotic entry decision in the *C. elegans* germline. (A) A simplified genetic pathway showing the proteins involved in regulating the proliferation *vs.* meiotic entry decision. LAG-2, which is expressed on the somatic DTC at the distal end of the gonad, interacts with the GLP-1/Notch receptor that is expressed on the germ cells. This is thought to cause the intracellular portion of GLP-1, ICD, to translocate to the nucleus and interact with the LAG-1 transcription factor, turning on genes necessary for proliferation. GLP-1/Notch signaling inhibits the activities of two redundant pathways (GLD-1 and GLD-2 pathways), through the activity of two PUF proteins, FBF-1 and FBF-2. The GLD-1 and GLD-2 pathways promote meiotic entry and/or inhibit the proliferative fate. (B) Gene model of *puf-8* illustrating the positions of the exons (boxes) and introns (connecting lines), as well as the 5′ and 3′ UTRs (black boxes) and the region encoding the eight PUF domain repeats (gray box). Also shown is the location of the *puf-8(oz192)* lesion, which results in a premature stop codon in the second PUF repeat, and the deletion associated with the *puf-8(q725)* allele.

Pumilio homologs, or PUF proteins (Pumilio and FBF), are a family of conserved proteins that bind RNA and affect the translation, stability, and/or localization of target mRNAs (Quenault *et al.* 2011; Wickens *et al.* 2002). *C. elegans* contains multiple PUF proteins, including FBF-1 and FBF-2, which participate in various aspects of germline development and maintenance. For example, in addition to their role in promoting the proliferative fate of GSCs, FBF-1 and FBF-2 promote the switch from spermatogenesis to oogenesis in germline sex determination by inhibiting the activity of *fem-3* (Zhang *et al.* 1997). PUF-3/11 control the growth of oocytes, whereas another group of PUF proteins, PUF-5/6/7, control the formation of oocytes (Hubstenberger *et al.* 2012; Lublin and Evans 2007). PUF-8 has multiple functions in germline development, including inhibiting dedifferentiation of primary spermatocytes, functioning redundantly with FBF-1 to promote the sperm/oocyte switch, and functioning redundantly with the KH-domain containing protein MEX-3 to promote mitosis in the GSCs (Ariz *et al.* 2009; Bachorik and Kimble 2005; Mainpal *et al.* 2011; Subramaniam and Seydoux 2003). Here, we demonstrate that PUF-8 also functions within the *C. elegans* germline to inhibit the proliferative fate of GSCs.

## Materials and Methods

### Nematode strains, nomenclature, and general methods

All strains were maintained using standard methods on nematode growth medium plates seeded with *Escherichia coli* OP50 (Brenner 1974). Strains were grown at 20° unless otherwise noted. Most strains were derived from wild-type Bristol strain N2. The following alleles were used in this study: LGI: *gld-2(q497)*, *ccIs4251[myo-3*::*Ngfp-lacZ*; *myo-3*::*Mtgfp] unc-15(e73)*, *gld-1(q485)*, LGII: *bli-2(e768)*, *lin-4(e912)*, *dpy-10(e128)*, *gld-3(q730)*, *puf-8(oz192*, *q725)*, *rol-6(e187)*, *nos-3(oz231)*, *unc-4(e120)*, LGIII: *unc-32(e189)*, *lin-12(ar170*, *q269)*, *glp-1(ar202*, *bn18*, *oz264*, *oz112oz120*, *q231*, *q175)*, *spe-6(hc49)*, *unc-25(e156)*, LGIV: *unc-24(e138)*, *fem-3(e1996)*, *dpy-20(e1282)*, *arIs51[cdh-3*::*gfp]*.

### Mapping and cloning of *teg-2*

Single-nucleotide polymorphism (SNP) mapping was performed using the Hawaiian CB4856 *(HA-8)* strain. From *teg-2(oz192) rol-6(e187)/HA-8*; *glp-1(ar202gf)* and *bli-2(e768) teg-2(oz192)/HA-8*; *glp-1(ar202gf)* animals, we identified 24 roller nontumorous and 34 blister nontumorous recombinants, respectively. The furthest roller nontumorous recombinants to the left of *rol-6(e187)* (*HA-8 rol-6(e187)*; *glp-1(ar202gf)*) extended to SNP uCE2-1737 and the furthest blister nontumorous recombinants to the right of *bli-2(e768)* (*bli-2(e768) HA-8*; *glp-1(ar202gf)*) extended to SNP F32A5[2] (supporting information, Figure S1, second map). This narrowed the critical region containing *teg-2* to a 98-kb region containing 18 genes (Figure S1, third map). Sequencing of one these genes, *puf-8*, revealed a G937T transversion (Figure S1, bottom gene model).

### *puf-8*::*gfp*::*tap* Mos1-mediated single-copy insertion (MosSCI)-integrated array

MosSCI was used to transform EG5003 [*unc-119(ed3) III*; *cxTi10882 IV*] animals with the 14.3-kb pDH173 construct [*puf-8 promoter*::*puf-8 genomic*::*gfp*::*tap*::*puf-8 3′UTR*, *C. briggsae unc-119(+)*] using the ‘direct method’ previously reported (Frokjaer-Jensen *et al.* 2008). The pDH173 construct was generated through modification of the pGB2 construct [*puf-8 promoter*::*puf-8 genomic*::*gfp*::*unc-54 3′UTR]* (Walser *et al.* 2006). First, the *unc-54 3′UTR* was replaced with the *puf-8 3′UTR* using SOEing polymerase chain reaction (Horton 1993). Finally, the *puf-8 promoter*::*puf-8 genomic*::*gfp*::*tap*::*puf-8 3′UTR* sequence was inserted into the MosSCI targeting plasmid, pCFJ178 (Frokjaer-Jensen *et al.* 2008), generating pDH173.

### Immunohistochemisty

Gonad dissection, fixation, and antibody staining were performed as previously described (Jones *et al.* 1996). Dissected gonads were fixed using 3% formaldehyde, 0.1 KPO4 (pH 7.2), for 10 min. The gonads were then postfixed in −20° 100% methanol for at least 10 min. Fixed gonads were incubated with 100 ng/mL of 4,6-diamidino-2-phenylindole hydrochloride (DAPI) in 1× phosphate-buffered saline for 5 min to visualize nuclear morphology. Rat anti-REC-8 (Pasierbek *et al.* 2001) (1:150 dilution) and Rabbit anti-HIM-3 (Zetka *et al.* 1999) (1:500 dilution)-specific antibodies were used to detect mitotic and meiotic cells, respectively. Mouse anti-GFP antibodies (3E6; Molecular Probes, 1:750 dilution) were used to detect green fluorescent protein (GFP) in the integrated transgenic PUF-8::GFP line (*ugSi1*). The 2° antibodies that were used include Donkey anti-rat Alexa488 (Molecular Probes, 1:200 dilution), Donkey anti-rabbit Alexa594 (Molecular Probes, 1:500 dilution) and Donkey anti-mouse Alexa488 (Molecular Probes, 1:200 dilution). Images, both differential interference contrast and fluorescent, were captured using a Zeiss Imager Z1 microscope equipped with an Axiocam MrM digital camera (Ziess). Images were viewed and analyzed using the AxioVision software. Photoshop software was used to assemble whole gonad arms from individual images.

## Results

### *teg-2(oz192)* enhances *glp-1(gf)* mutations and suppresses a *glp-1(lf)* mutation

To identify additional regulators functioning in the proliferative fate *vs.* meiotic entry decision, a mutant screen was conducted for mutations that enhance the overproliferative phenotype of the weak *glp-1* gain-of-function allele, *glp-1(oz112oz120gf)*, resulting in germline tumor formation (Wang *et al.* 2012). We have previously shown that two genes identified in this screen, *teg-1* and *teg-4* (tumorous enhancer of *glp-1(oz112oz120)*), encode likely splicing factors (Mantina *et al.* 2009; Wang *et al.* 2012). Here, we describe the characterization and cloning of another gene identified in this screen, *teg-2*. We demonstrate that *teg-2* is allelic to *puf-8*, which encodes an RNA binding Pumilio homolog (Wickens *et al.* 2002); therefore, we will refer to *teg-2* as *puf-8* throughout this paper.

**Figure 2:**
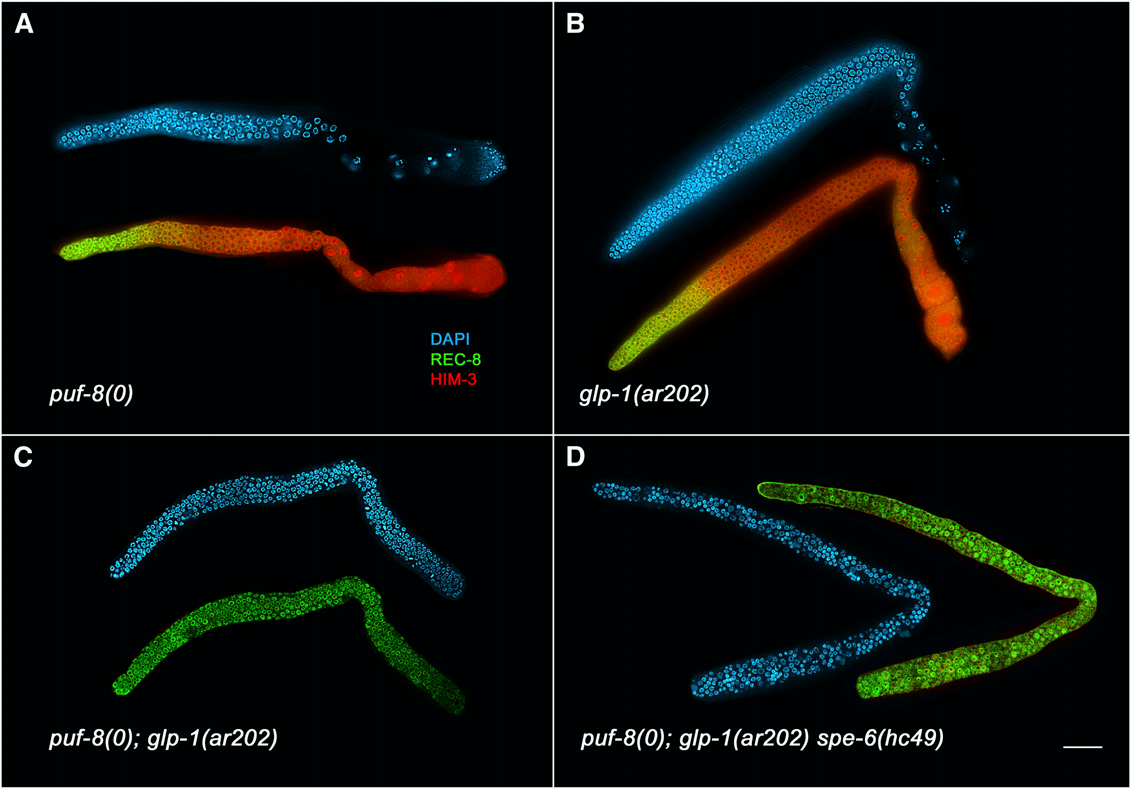
*puf-8(0)* enhances germline overproliferation of *glp-1(gf)*. Dissected hermaphrodite gonads were fixed and stained with DAPI (blue) to visualized nuclear morphology, anti-REC-8 antibodies (green) to mark mitotic cells and anti-HIM-3 antibodies (red) to mark meiotic cells. Animals were grown at 15° and dissected 1 d after L4. Both *puf-8(oz192)* (A) and *glp-1(ar202)* (B) single mutants contain proliferative cells in the distal end of the gonad (left), followed by meiotic cells and gametes. In contrast, the *puf-8(oz192)*; *glp-1(ar202)* double mutant (C) contains only proliferative cells. (D) *spe-6(hc49)* does not suppress the *puf-8(oz192)* tumor, suggesting that the over-proliferation is not due to dedifferentiation of male germ cells. Scale bar = 40 microns.

Although *puf-8* single mutants show no germline overproliferation due to a defect in the proliferative fate *vs.* meiotic entry decision (Bachorik and Kimble 2005; Subramaniam and Seydoux 2003), *puf-8(0)* is a strong enhancer of *glp-1(gf)* in both males and hermaphrodites (Figure 2; Table S1). *puf-8(0)*; *glp-1(gf)* animals have completely tumorous germlines, with no evidence of any cells entering into meiotic prophase, even at the 15° permissive temperature. This enhancement is not specific to the *glp-1* allele because *puf-8(0)* enhances multiple *glp-1(gf)* alleles (Table 1). In addition, *puf-8(0)* enhances when only one copy of *glp-1(gf)* is present (Table 1), further suggesting that *puf-8(0)* is a strong enhancer of *glp-1(gf)*. Furthermore, loss of *puf-8* activity partially suppresses the premature meiotic entry phenotype of the *glp-1(bn18)* partial loss-of-function allele (Table 2). The interaction between *puf-8(0)* and both loss and gain-of-function alleles of *glp-1* suggests that *puf-8* functions as an inhibitor of the proliferative fate and/or promoter of the meiotic fate.

**Table 1:** *puf-8(0)* enhances the overproliferation phenotype of *glp-1(gf)* at 15°

Genotype*^a^*	Wild-type, %*^b^*	Complete Tumor, %*^c^*	Incomplete Tumor, %*^d^*	n*^e^*
*puf-8(oz192)*	100	0	0	23
*puf-8(q725)*	100	0	0	36
*glp-1(ar202)*	100	0	0	10
*glp-1(oz264)**^f^*	100	0	0	9
*puf-8(oz192)*; *glp-1(ar202)*	0	100	0	35
*puf-8(q725)*; *glp-1(ar202)*	0	100	0	35
*puf-8(oz192)*; *glp-1(ar202)/+**^g^*	0	74	26	27
*puf-8(oz192)*; *glp-1(oz264)*	0	100	0	23
*puf-8(q725)*; *glp-1(oz264)*	0	98	2	54

a All animals maintained at 15°. For each genotype, gonads were dissected and stained with DAPI, anti-REC-8 antibodies, and anti-HIM-3 antibodies. All animals were dissected at 1 d after the fourth larval stage.

b A gonad arm was scored as wild-type if no overproliferation was observed.

c A gonad arm with a complete tumor contained no anti-HIM-3(+) cells or fully differentiated cells. Rather, all cells were anti-REC-8(+).

d A gonad arm with an incomplete tumor contained extensive over-proliferation throughout the gonad arm; however, small pockets of anti-HIM-3(+) cells, or sperm, were observed.

e Number of dissected gonad arms analyzed.

f The GLP-1(oz264) protein contains a G to E amino acid substitution at amino acid 528 of the GLP-1 protein (Kerins *et al.* 2010).

g Actual genotype *puf-8(oz192) unc-4(e120)/puf-8(oz192); glp-1(ar202)/+*.

**Table 2:** *puf-8(0)* suppresses the Glp phenotype of a weak *glp-1(ts)* allele

Genotype	Temp.*^a^*	Glp, %*^b^*	n*^c^*
*glp-1(bn18)*	22°	20	45
*puf-8(oz192)*; *glp-1(bn18)*	22°	3	141
*puf-8(q725)*; *glp-1(bn18)*	22°	0.4	488
*glp-1(bn18)*	25°	100	15
*puf-8(oz192)*; *glp-1(bn18)*	25°	96	17
*puf-8(q725)*; *glp-1(bn18)*	25°	70	110

a Homozygous strains were used and maintained at 15° (permissive temperature) then shifted to the test restrictive temperature as L4 animals and allowed to self-fertilize. Progeny were scored 3 d later by Nomarski optics, after having grown to adulthood at the restrictive temperature.

b Germline proliferation defective (Glp); animals lacked a distal proliferative zone resulting in only sperm being present in the gonad arm.

c Number of gonad arms analyzed.

### *teg-2* and *puf-8* are allelic

We used SNP mapping to narrow the critical region containing *teg-2* to a 98-kb region on chromosome II containing 18 genes. Sequencing of one of these genes, *puf-8*, revealed a G to T transversion at position 937 from the initiator ATG. This mutation is predicted to cause a premature amber stop codon in the second of the eight PUF repeats; therefore, the mutation likely results in a strong loss-of-function or null allele. To determine whether *teg-2* and *puf-8* are allelic, we performed a complementation test using the *puf-8(q725)* deletion allele (Bachorik and Kimble 2005) and found that *puf-8(q725)/teg-2(oz192) rol-6(e187)*; *glp-1(ar202*gf*)* animals have a tumorous germline at 15° (n > 20). Therefore, *teg-2* is allelic with *puf-8*, which encodes a homolog of *Drosophila* Pumilio (Figure S1).

### *puf-8(0)*; *glp-1(gf)* tumor is due to disruption of proliferation *vs.* meiotic entry

*puf-8* animals were previously shown to have an incompletely penetrant male germ cell meiotic progression phenotype, such that in ~44% of animals grown at 25°, primary spermatocytes dedifferentiate, exiting from meiosis and re-entering mitosis, resulting in the formation of a tumor in the proximal end of the gonad (Subramaniam and Seydoux 2003). Therefore, the tumor enhancement we observe in a *glp-1(gf)* background may not be due to a defect in the proliferative fate *vs.* meiotic entry decision but rather may be a result of dedifferentiation of male germ cells that fail to properly progress through meiotic prophase. However, we found that *puf-8(0)* enhances germline overproliferation in *glp-1(gf)* animals that have a feminized germline (33/33 *puf-8(oz192)*; *glp-1(ar202)*; *fem-3(e1996)/fem-3(e1996)*; or *fem-3(+)* gonad arms were completely tumorous, with one-third of the gonad arms predicted to be homozygous for *fem-3(e1996)*; Figure S2). It was also previously demonstrated that the dedifferentiation phenotype of *puf-8(0)* animals could be suppressed if meiotic progression in male germ cells is stalled (Subramaniam and Seydoux 2003), such as the stalling that occurs in a *spe-6* mutant (Varkey *et al.* 1993). We found that that *puf-8(q725)*; *glp-1(ar202*gf*) spe-6(hc49)* animals have a tumorous germline, even though *spe-6(hc49)* would suppress a dedifferentiation tumor (Figure 2). Furthermore, at various stages of larval development and adulthood, all germ cells in *puf-8(0)*; *glp-1(gf)* animals are mitotic, with no evidence of entering into meiotic prophase and then dedifferentiating (Table 1; *puf-8(0)*; *glp-1(ar202)* dissected gonads were all anti-REC-8(+) and anti-HIM-3(−) at both the late L2 and late L3 stages, n = 18 for each). Therefore, cells are unlikely to have entered into meiosis early in development and then dedifferentiated, resulting in a tumor in adult animals. We conclude that the tumor observed in *puf-8(0)*; *glp-1(gf)* animals is not due to dedifferentiation of male germ cells, but rather due to a disruption of the proliferative fate *vs.* meiotic entry balance.

### *puf-8* functions upstream of, or parallel to, the *glp-1/Notch* signaling pathway

To better understand how *puf-8* may be regulating the proliferative fate *vs.* differentiation decision, we sought to determine where *puf-8* may function in the genetic pathway regulating this decision by performing genetic epistasis experiments. Because *puf-8* appears to inhibit the proliferative fate and/or promote meiotic entry, it is possible that *puf-8* functions in either the *gld-1* or *gld-2* pathways, which also function to inhibit the proliferative fate and/or promote meiotic entry (Figure 1). Because the *gld-1* and *gld-2* pathways function redundantly, if *puf-8* were to function in either of these pathways, *puf-8(0)* would likely form a synthetic tumor when the function of the other pathway, not containing *puf-8*, was eliminated. We found that animals lacking *puf-8* activity do not form synthetic tumors when the activities of any of the genes in the *gld-1* or *gld-2* pathways are also eliminated (Table 3), suggesting that *puf-8* likely does not function in either of these two pathways. It is possible that *puf-8* may have a minor role in either the *gld-1* or *gld-2* pathways and that reducing its function does not reduce pathway function enough to form a synthetic tumor when the activity of the other pathway is also reduced. However, given that *puf-8(0)* appears to be a stronger enhancer of *glp-1(gf)* than any of the known components of the *gld-1* and *gld-2* pathways, we consider this unlikely. Therefore, *puf-8* may function as a negative regulator of GLP-1/Notch signaling, or parallel to this signaling pathway.

**Table 3:** *puf-8* is unlikely to function in the *gld-1* or *gld-2* pathways

Genotype	Length Proliferative Zone (Cell Diameter, Range)*^a^*
Wild type (N2)	20 (15-24)
*puf-8(oz192)*	13 (7−18)
*gld-1(q485)*	14 (10-18)*^b^*
*gld-2(q497)*	27 (23-33)*^c^*
*gld-3(q730)*	27 (23-32)*^d^*
*nos-3(oz231)*	20 (14-24)*^c^*
*gld-1(q485)*; *puf-8(oz192)*	14 (11-16)*^e^*
*gld-2(q497)*; *puf-8(oz192)*	19 (15-22)
*gld-3(q730) puf-8(oz192)*	18 (16-20)
*puf-8(oz192) nos-3(oz231)*	14 (11-17)

a Animals were dissected 1 d past the L4 stage and gonads were fixed and stained with DAPI, anti-REC-8 antibodies and anti-HIM-3 antibodies to visualize nuclear morphology and to determine the extent of proliferation *vs.* meiotic entry. The length of the proliferative zone for each double mutant was counted for a minimum of five gonad arms to obtain an average. The range of results obtained is shown. At least 20 additional gonad arms were analyzed in each double mutant, none of which showed evidence of over-proliferation.

b In *gld-1(q485)* animals, germ cells enter into meiosis normally; however, female germ cells are unable to complete meiosis. Rather, these cells re-enter the mitotic cell cycle, forming a tumor in the proximal end of the gonad. Data obtained from Eckmann *et al.* 2004.

c Data obtained from Hansen *et al.* 2004b.

d Data obtained from Eckmann *et al.* 2004.

e Whereas *gld-1(q485)* single mutants are feminized, with female germ cells failing to progress through meiotic prophase, instead returning to the mitotic cell cycle and forming a proximal tumor, *gld-1(q485)*; *puf-8(oz192)* animals make sperm. Therefore, *puf-8(oz192)* suppresses *gld-1(q485)* germline feminization. A total of 76% (n = 35) of gonad arms also contained proximal proliferation typical of *gld-1(q485)* single mutants. *gld-1(q485)*; *puf-8(q725)*; *glp-1(q175)* are also Glp (n = 72), further supporting that the proximal proliferation in the 76% of gonad arms is due to de-differentiation rather than a synthetic interaction between *gld-1* and *puf-8*.

To determine whether *puf-8* is a general negative regulator of Notch signaling, we tested for an interaction with the Notch signaling pathway in other Notch-regulated cell fate decisions, including the anchor cell *vs.* ventral uterine precursor cell decision, and the redundant L1 arrest phenotype of *lin-12(q269) glp-1(231)* animals [LIN-12 is the other Notch receptor in *C. elegans* and functions redundantly with GLP-1 in certain developmental processes (Austin and Kimble 1989; Yochem and Greenwald 1989)]. We found no interaction between *puf-8* and the Notch signaling pathway in either of these contexts (Table S2), suggesting that *puf-8* is not a general negative regulator of Notch signaling but rather may function specifically in the germline to negatively regulate GLP-1/Notch signaling or function in parallel to the GLP-1/Notch signaling pathway to inhibit the proliferative fate.

### PUF-8 is expressed throughout the entire proliferative zone and in early meiotic cells

**Figure 3:**
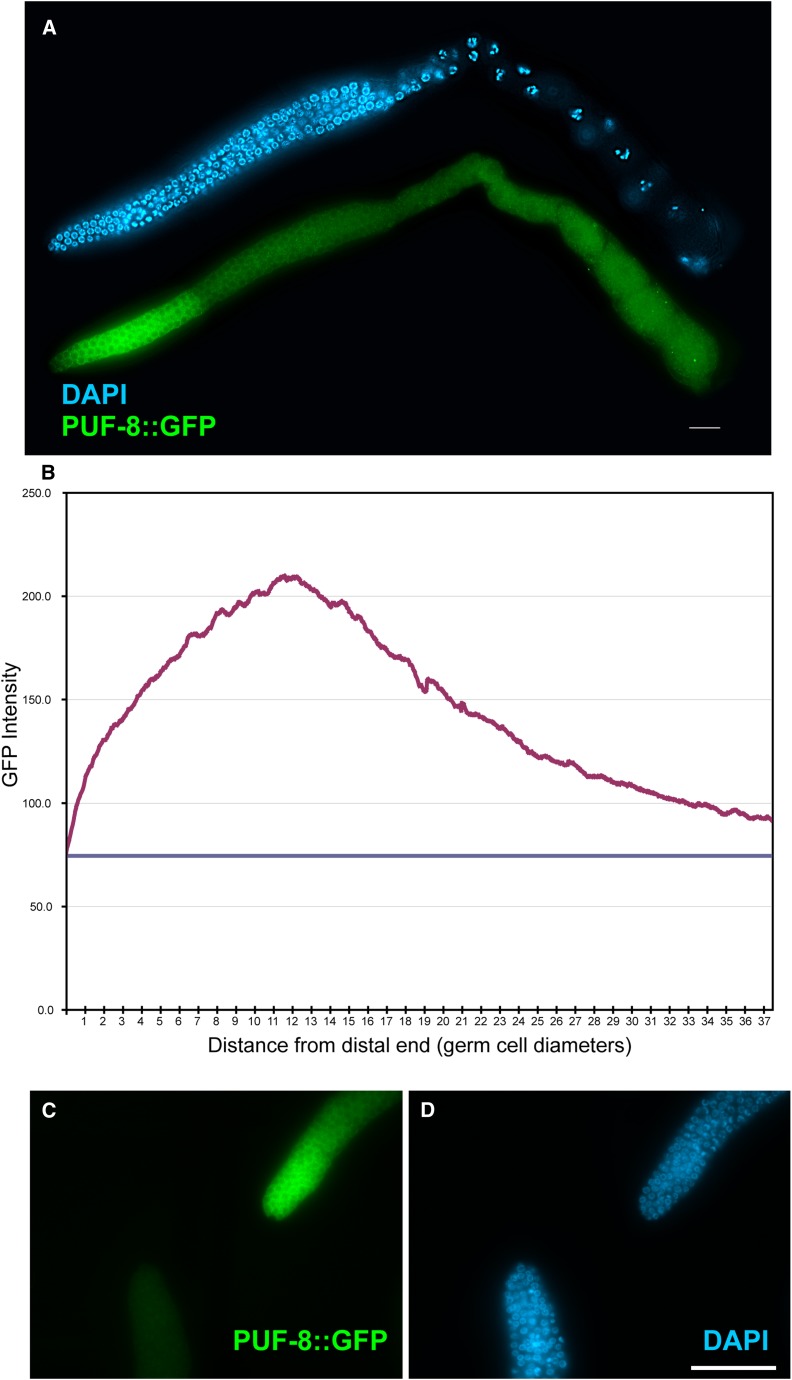
Expression pattern of PUF-8 in the proliferative zone. (A) Dissected *puf-8(q725)*; *ugSi1[puf-8*::*gfp]* hermaphrodite gonad arm 1 d after L4 stained with DAPI (blue) and anti-GFP antibodies (green). Scale bar = 20 microns. (B) Graph of the intensity of the PUF-8::GFP accumulation in the proliferative zone average from 10 gonad arms. Horizontal blue line is the level of background staining as measured in wild-type gonad arms stained with anti-GFP antibodies (n = 10). The x-axis is the distance from the distal end of the gonad arm as measured in germ cell diameters. The y-axis is the GFP intensity in arbitrary units, which was measured based on pixel intensity using ImageJ software (NIH). *ugSi1[puf-8*::*gfp]* is an integrated single copy insertion using the pDH173 plasmid, which is a modification of the pGB2 plasmid (Walser *et al.* 2006), which was modified to include a TAP tag (HA::8xHis::Myc) after the GFP domain, and replacing the *unc-54* 3′UTR with the *puf-8* 3′UTR. The construct was inserted into the *cxTi10882* site on chromosome IV. Similar staining patterns were observed with the IS30 strain, which carries an integrated *puf-8*::*gfp* construct obtained through bombardment. The *ugSi1* strain is rescuing, fully rescuing the dedifferentiation and small germline phenotype of *puf-8(q725)* at 25°C (n = 35), and 83% of *puf-8(q725)*; *glp-1(ar202)*; *ugSi1* animals containing differentiated cells (n = 52), whereas no *puf-8(q725)*; *glp-1(ar202)* animals have differentiated cells in the gonad (n = 35). The distal proliferative zone length was only partially rescued, still being ~3 cell diameters shorter (16 ± 2.4, n = 15) than wild-type (19 ± 3.6, n = 17), but ~3 cell diameters longer than *puf-8(q725)* (13 ± 1.1, n = 11). (C and D) *puf-8(q725)*; *ugSi1* (top) and wild-type (bottom) gonad arm stained with anti-GFP antibodies (C; green) and DAPI (D; blue), in the same field of view showing intensity of PUF-8::GFP staining over background. Scale bar = 50 microns.

The PUF-8 protein was previously shown to be enriched in the distal end of the gonad (Ariz *et al.* 2009); however, it was unclear as to its spatial distribution pattern relative to where cells show signs of entering meiotic prophase. To determine whether its spatial distribution in the distal end could help explain how PUF-8 may be functioning in regulating the proliferative fate *vs.* meiotic entry decision, we measured the accumulation of PUF-8::GFP relative to the progression of cells from the proliferative fate to meiotic entry. We constructed an integrated single copy *puf-8*::*gfp*::*tap* transgenic line (*ugSi1*), which largely rescues multiple *puf-8(0)* phenotypes, and found that PUF-8::GFP is expressed throughout the distal end of the gonad, with slightly lower levels within the first third to half of the proliferative zone (Figure 3). Protein levels gradually decrease proximally until eventually plateauing at ~40 cell diameters, well beyond where all cells normally have entered into meiotic prophase (Crittenden *et al.* 1994; Hansen *et al.* 2004a). Within the proliferative zone, ~30% to 40% of germ cells are in premeiotic S-phase (Fox *et al.* 2011). Therefore, assuming that these ~30% to 40% premeiotic cells are located in the more proximal end of the proliferative zone, PUF-8 is at its maximum expression level in the approximate region of the proliferative zone where cells are likely entering pre-meiotic S-phase, consistent with a role in facilitating the switch from proliferation to meiosis.

## Discussion

**Figure 4:**
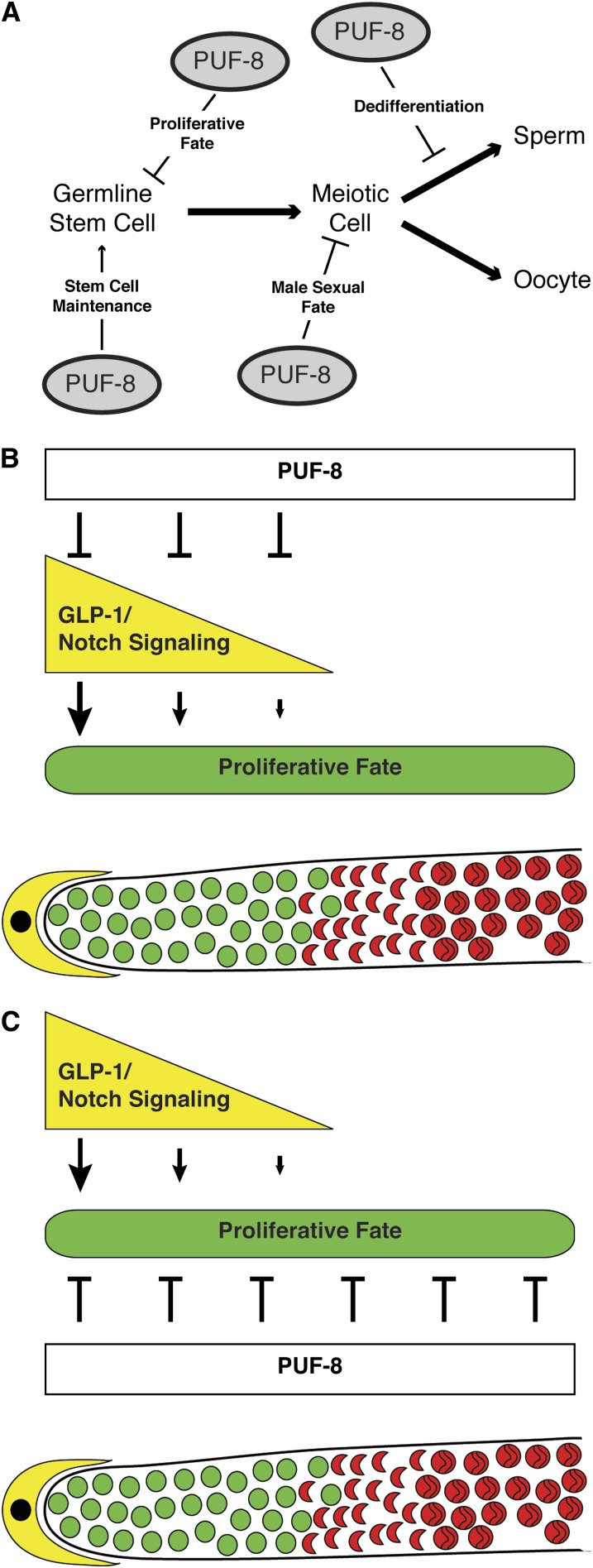
Models of PUF-8 function in gamete development. (A) PUF-8 functions at multiple stages in the development of gametes. These functions include promoting stem cell maintenance (Ariz *et al.* 2009; Bachorik and Kimble 2005), inhibition of the proliferative fate in GSCs (this work), inhibition of the male sexual fate (Bachorik and Kimble 2005), and inhibition of dedifferentiation of male germ cells progressing through meiosis (Subramaniam and Seydoux 2003). (B and C) Models of PUF-8 function in regulating the balance between the proliferative fate and meiotic entry. Illustration of distal end of gonad showing the DTC (yellow), mitotic cells (green) and meiotic cells (red). (B) PUF-8 may inhibit the activity of GLP-1/Notch signaling. GLP-1/Notch signaling levels are thought to be high at the very distal end, and lower as cells progress proximally. In this model, PUF-8 contributes to the lowering of GLP-1/Notch signaling levels, allowing cells to enter meiotic prophase. The inhibition of GLP-1/Notch signaling may not be direct, but rather PUF-8 may inhibit the activity of a positive regulator of GLP-1/Notch signaling. (C) PUF-8 may inhibit the proliferative fate in a pathway that is parallel to the GLP-1/Notch signaling pathway. In both models (B and C) PUF-8 is shown to be active throughout the distal end of the gonad; however, it is possible that another factor or post-translational modification causes PUF-8 not to be active in some regions where it is expressed.

A defining feature of stem cells is their ability to produce both self-renewing and differentiating daughter cells. For GSCs, a disruption in the balance between self-renewal (proliferation) and differentiation (meiotic entry) can have detrimental effects on the reproductive fitness of the animal. Therefore, animals have evolved robust systems to regulate the balance between the proliferative fate and meiotic entry, including redundant pathways and modulating factors. Although the GLP-1/Notch signaling pathway is the major regulator of this balance in the *C. elegans* germline, other factors have been identified that modulate this pathway, work parallel to it, or are regulated by it (Hansen and Schedl 2013; Kimble and Crittenden 2007). We have identified the PUF protein, PUF-8, as an additional player in regulating this balance. PUF-8 inhibits the proliferative fate in the *C. elegans* germline, either through inhibition of the GLP-1/Notch signaling pathway, or functioning parallel to it (Figure 4).

### PUF proteins and the maintenance of stem cells

Maintenance of stem cells by PUF proteins has been found in a number of other systems. In Drosophila, Pumilio is expressed in GSCs, and its absence causes female GSCs to prematurely differentiate as cystoblasts (Forbes and Lehmann 1998; Lin and Spradling 1997). In mouse hematopoietic stem cells, both Pum1 and Pum2 are highly transcribed (Spassov and Jurecic 2003). Mouse Pum2 is also highly expressed in male germ cells and, when mutated, *pum2(0)* animals have significantly smaller testes (Xu *et al.* 2007). In addition, in the *C. elegans* germline, the two closely related PUF proteins, FBF-1 and FBF-2, function downstream of GLP-1/Notch signaling to promote the proliferative fate in adult animals (Figure 1) (Crittenden *et al.* 2002). Therefore, Pumilio proteins appear to have conserved functions in regulating stem cell behavior. However, most PUF proteins that have been implicated in regulating GSC behavior appear to promote the proliferative fate. Here, we have demonstrated that PUF-8 also functions in the opposite direction, to inhibit the proliferative fate. We have demonstrated that *puf-8* is unlikely to function in either the *gld-1*− and *gld-2*−redundant pathways that function downstream of *glp-1* signaling. Therefore, *puf-8* is likely to function upstream of these pathways, possibly as a negative regulator of GLP-1/Notch signaling, or in a pathway that functions parallel to GLP-1/Notch signaling (Figure 4, B and C). None of the core components of *glp-1* signaling contain a canonical PUF-8 binding site in their 3′ UTRs (Stumpf *et al.* 2008), and none of these components were identified in a immunoprecipitation of potential PUF-8 mRNA targets (Mainpal *et al.* 2011); therefore, if *puf-8* does inhibit *glp-1* signaling, it likely does so indirectly, possibly by inhibiting the activity of a positive regulator.

Whether PUF-8 functions to inhibit the activity of the GLP-1/Notch signaling pathway or functions in a parallel pathway, its expression throughout the proliferative zone, and well past the region where all cells have entered into meiotic prophase (Figure 3), suggests a model in which PUF-8 dampens the overall signal promoting the proliferative fate. In this model, in the very distal end of the gonad where the germ cells are close to the distal tip cell (DTC) and GLP-1/Notch signaling levels are thought to be at their peak, this high level of signaling is able to overcome any inhibition of the proliferative fate provided by PUF-8. However, as cells move more proximally, the DTC is less able to promote high GLP-1/Notch signaling levels, and PUF-8 continues to inhibit the proliferative fate, either through contributing to the decrease in GLP-1/Notch signaling levels (Figure 4B) or by inhibiting the proliferative fate independent of GLP-1/Notch signaling (Figure 4C).

Although these models take into account the expression of PUF-8 throughout much of the distal end of the gonad, they also assume that PUF-8 is active in all areas where it is expressed. It remains possible that PUF-8 activity is controlled through a post-translational modification, or through the binding of a spatially regulated cofactor, which limits PUF-8’s inhibition of the proliferative fate to cells in the region of the distal gonad where they enter meiotic prophase. Indeed, PUF proteins in other systems work cooperatively with other proteins to regulate target gene activity. For example, the Drosophila protein Nanos works with Pumilio to repress the translation of *hunchback* mRNA during embryonic patterning (Barker *et al.* 1992; Tautz 1988). In this system, Pumilio is expressed throughout the embryo (Macdonald 1992), but is only active in the posterior end where Nanos protein is localized (Murata and Wharton 1995). Therefore, it is possible that PUF-8 is not active throughout the entire mitotic zone but rather is limited to certain regions due to the activity of another protein. The *nos-3* gene, which encodes a protein with similarity to Drosophila Nanos, has previously been shown to inhibit the proliferative fate and/or promote meiotic entry (Hansen *et al.* 2004b); however, *nos-3* appears to function in the *gld-1* pathway, whereas *puf-8* does not (Table 3). Therefore, if PUF-8 functions with another protein that spatially regulates its activity, this other protein likely is not NOS-3.

### PUF-8 inhibits the proliferative fate at two stages in germ cell progression

A portion of *puf-8(0)* single mutants were previously shown to form a germline tumor in the proximal end of their gonads (Subramaniam and Seydoux 2003). In animals that form a proximal tumor, GSCs enter the path to differentiation and enter into meiosis normally; however, some cells destined for the male fate fail to properly progress through meiotic prophase but rather dedifferentiate and reenter the mitotic cell cycle. Therefore, PUF-8 actively limits germ cell proliferation in the proximal gonad by preventing dedifferentiation of male germ cells undergoing spermatogenesis. We have demonstrated that *puf-8* also inhibits the proliferative fate in GSCs, prior to them entering into meiotic prophase. This inhibition is distinct from the previously described dedifferentiation inhibition because it inhibits the proliferative fate of cells that have not yet entered into meiotic prophase, and is irrespective of the eventual sex of the cells. Therefore, *puf-8* inhibits the proliferative fate in two distinct stages of germ cell development (Figure 4A).

It is not unique for a single factor to inhibit the proliferative fate in more than one step in the formation of a gamete. For example, *gld-1*, which encodes a KH-domain containing translational inhibitor (Jones and Schedl 1995), also inhibits the proliferative fate in *C. elegans* in two stages of germ cell development. Like *puf-8*, *gld-1* inhibits proliferation and/or promotes meiotic entry of GSCs (Francis *et al.* 1995b). In addition, like *puf-8*, *gld-1* inhibits dedifferentiation of cells that have entered meiotic prophase; however, while *puf-8* inhibits dedifferentiation of male germ cells (Subramaniam and Seydoux 2003), *gld-1* inhibits dedifferentiation of female germ cells (Francis *et al.* 1995a). Some of the cells within the *gld-1* single mutant tumor are positive for somatic cell markers, suggesting that they are differentiating, similar to cells in a mammalian teratoma (Biedermann *et al.* 2009; Ciosk *et al.* 2006). It is intriguing that two factors, which are both likely to regulate gene targets through mRNA translation and/or stability, inhibit the proliferative fate in, at least, two stages of germ cell development.

PUF-8 has other germline functions besides inhibition of the proliferative fate (Figure 4A), including promoting stem cell maintenance (Ariz *et al.* 2009; Bachorik and Kimble 2005; Mainpal *et al.* 2011) and inhibiting the male sexual fate in hermaphrodites (Bachorik and Kimble 2005). The PUF-8 homolog in the closely related nematode, *C. briggsae*, also functions in germline sex determination, although to promote sperm production (Beadell *et al.* 2011). *C. elegans* PUF-8 has also been shown to have a somatic role in vulval development (Walser *et al.* 2006). Therefore, PUF-8 likely has many mRNA targets, many of which will be specific to only one of PUF-8’s functions. A clear future direction to understanding how PUF-8 functions in regulating the proliferative fate *vs.* meiotic entry decision will be to identify the mRNA targets that are specifically involved in this decision.

## Supplementary Material

Supporting Information

## References

[bib1] Ariz M. Mainpal R. Subramaniam K., 2009 *C. elegans* RNA-binding proteins PUF-8 and MEX-3 function redundantly to promote germline stem cell mitosis. Dev. Biol. 326: 295–304 19100255 10.1016/j.ydbio.2008.11.024 PMC2680957

[bib2] Austin J. Kimble J., 1989 Transcript analysis of glp-1 and lin-12, homologous genes required for cell interactions during development of *C. elegans*. Cell 58: 565–571 2758467 10.1016/0092-8674(89)90437-6

[bib3] Bachorik J. L. Kimble J., 2005 Redundant control of the *Caenorhabditis elegans* sperm/oocyte switch by PUF-8 and FBF-1, two distinct PUF RNA-binding proteins. Proc. Natl. Acad. Sci. USA 102: 10893–10897 16037210 10.1073/pnas.0504593102 PMC1182444

[bib4] Barker D. D. Wang C. Moore J. Dickinson L. K. Lehmann R., 1992 Pumilio is essential for function but not for distribution of the Drosophila abdominal determinant Nanos. Genes Dev. 6: 2312–2326 1459455 10.1101/gad.6.12a.2312

[bib5] Beadell A. V. Liu Q. Johnson D. M. Haag E. S., 2011 Independent recruitments of a translational regulator in the evolution of self-fertile nematodes. Proc. Natl. Acad. Sci. USA 108: 19672–19677 22106259 10.1073/pnas.1108068108 PMC3241777

[bib6] Biedermann B. Wright J. Senften M. Kalchhauser I. Sarathy G., 2009 Translational repression of cyclin E prevents precocious mitosis and embryonic gene activation during *C. elegans* meiosis. Dev. Cell 17: 355–364 19758560 10.1016/j.devcel.2009.08.003

[bib7] Brenner S., 1974 The genetics of *Caenorhabditis elegans*. Genetics 77: 71–94 4366476 10.1093/genetics/77.1.71 PMC1213120

[bib8] Ciosk R. DePalma M. Priess J. R., 2006 Translational regulators maintain totipotency in the *Caenorhabditis elegans* germline. Science 311: 851–853 16469927 10.1126/science.1122491

[bib9] Crittenden S. L. Troemel E. R. Evans T. C. Kimble J., 1994 GLP-1 is localized to the mitotic region of the *C. elegans* germ line. Development 120: 2901 7607080 10.1242/dev.120.10.2901

[bib10] Crittenden S. L. Bernstein D. S. Bachorik J. L. Thompson B. E. Gallegos M., 2002 A conserved RNA-binding protein controls germline stem cells in *Caenorhabditis elegans*. Nature 417: 660 12050669 10.1038/nature754

[bib11] Eckmann C. Crittenden S. L. Suh N. Kimble J., 2004 GLD-3 and control of the mitosis/meiosis decision in the germline of *Caenorhabditis elegans*. Genetics 168: 147 15454534 10.1534/genetics.104.029264 PMC1448115

[bib12] Forbes A. Lehmann R., 1998 Nanos and Pumilio have critical roles in the development and function of Drosophila germline stem cells. Development 125: 679–690 9435288 10.1242/dev.125.4.679

[bib13] Fox P. M. Vought V. E. Hanazawa M. Lee M. H. Maine E., 2011 Cyclin E and CDK-2 regulate proliferative cell fate and cell cycle progression in the *C. elegans* germline. Development 138: 2223–2234 21558371 10.1242/dev.059535 PMC3091494

[bib14] Francis R. Barton M. K. Kimble J. Schedl T., 1995 a gld-1, a tumor suppressor gene required for oocyte development in *Caenorhabditis elegans*. Genetics 139: 579–606 7713419 10.1093/genetics/139.2.579 PMC1206368

[bib15] Francis R. Maine E. Schedl T., 1995 b Analysis of the multiple roles of gld-1 in germline development: interactions with the sex determination cascade and the glp-1 signaling pathway. Genetics 139: 607–630 7713420 10.1093/genetics/139.2.607 PMC1206369

[bib16] Frokjaer-Jensen C. Davis M. W. Hopkins C. E. Newman B. J. Thummel J. M., 2008 Single-copy insertion of transgenes in *Caenorhabditis elegans*. Nat. Genet. 40: 1375–1383 18953339 10.1038/ng.248 PMC2749959

[bib17] Hansen D. Schedl T., 2006 The regulatory network controlling the proliferation-meiotic entry decision in the Caenorhabditis elegans germ line. Curr. Top. Dev. Biol. 76: 185–215 17118267 10.1016/S0070-2153(06)76006-9

[bib18] Hansen D. Schedl T., 2013 Stem cell proliferation vs. meiotic fate decision in Caenorhabditis elegans. Adv. Exp. Med. Biol. 757: 71–99 22872475 10.1007/978-1-4614-4015-4_4 PMC3786863

[bib19] Hansen D. Hubbard E. J. Schedl T., 2004 a Multi-pathway control of the proliferation *vs.* meiotic development decision in the *Caenorhabditis elegans* germline. Dev. Biol. 268: 342–357 15063172 10.1016/j.ydbio.2003.12.023

[bib20] Hansen D. Wilson-Berry L. Dang T. Schedl T., 2004 b Control of the proliferation *vs.* meiotic development decision in the *C. elegans* germline through regulation of GLD-1 protein accumulation. Development 131: 93–104 14660440 10.1242/dev.00916

[bib21] Horton R. M., 1993 In vitro recombination and mutagenesis of DNA: SOEing together tailor-made genes. Methods Mol. Biol. 15: 251–261 21400283 10.1385/0-89603-244-2:251

[bib22] Hubstenberger A. Noble S. L. Cameron C. Shtofman R. Gutman S., 2012 A network of PUF proteins and Ras signaling promote mRNA repression and oogenesis in *C. elegans*. Dev. Biol. 366: 218–231 22542599 10.1016/j.ydbio.2012.03.019 PMC3361503

[bib23] Jones A. R. Schedl T., 1995 Mutations in gld-1, a female germ cell-specific tumor suppressor gene in *Caenorhabditis elegans*, affect a conserved domain also found in Src-associated protein Sam68. Genes Dev. 9: 1491–1504 7601353 10.1101/gad.9.12.1491

[bib24] Jones A. R. Francis R. Schedl T., 1996 GLD-1, a cytoplasmic protein essential for oocyte differentiation, shows stage- and sex-specific expression during *Caenorhabditis elegans* germline development. Dev. Biol. 180: 165 8948583 10.1006/dbio.1996.0293

[bib25] Kadyk L. C. Kimble J., 1998 Genetic regulation of entry into meiosis in *Caenorhabditis elegans*. Development 125: 1803–1813 9550713 10.1242/dev.125.10.1803

[bib26] Kerins J. A. Hanazawa M. Dorsett M. Schedl T., 2010 PRP-17 and the pre-mRNA splicing pathway are preferentially required for the proliferation *vs.* meiotic development decision and germline sex determination in Caenorhabditis elegans. Dev. Dyn. 239: 1555–1572 20419786 10.1002/dvdy.22274 PMC3097115

[bib27] Killian D. J. Hubbard E. J., 2005 *Caenorhabditis elegans* germline patterning requires coordinated development of the somatic gonadal sheath and the germ line. Dev. Biol. 279: 322–335 15733661 10.1016/j.ydbio.2004.12.021

[bib28] Kimble J. Crittenden S. L., 2007 Controls of germline stem cells, entry into meiosis, and the sperm/oocyte decision in *Caenorhabditis elegans*. Annu. Rev. Cell Dev. Biol. 23: 405–433 17506698 10.1146/annurev.cellbio.23.090506.123326

[bib29] Lamont L. B. Crittenden S. L. Bernstein D. Wickens M. Kimble J., 2004 FBF-1 and FBF-2 regulate the size of the mitotic region in the *C. elegans* germline. Dev. Cell 7: 697–707 15525531 10.1016/j.devcel.2004.09.013

[bib30] Lin H. Spradling A. C., 1997 A novel group of pumilio mutations affects the asymmetric division of germline stem cells in the Drosophila ovary. Development 124: 2463–2476 9199372 10.1242/dev.124.12.2463

[bib31] Lublin A. L. Evans T. C., 2007 The RNA-binding proteins PUF-5, PUF-6, and PUF-7 reveal multiple systems for maternal mRNA regulation during *C. elegans* oogenesis. Dev. Biol. 303: 635–649 17234175 10.1016/j.ydbio.2006.12.004

[bib32] Macdonald P. M., 1992 The Drosophila pumilio gene: an unusually long transcription unit and an unusual protein. Development 114: 221–232 1576962 10.1242/dev.114.1.221

[bib33] Mainpal R. Priti A. Subramaniam K., 2011 PUF-8 suppresses the somatic transcription factor PAL-1 expression in *C. elegans* germline stem cells. Dev. Biol. 360: 195–207 21968099 10.1016/j.ydbio.2011.09.021 PMC3736097

[bib34] Mantina P. Macdonald L. Kulaga A. Zhao L. Hansen D., 2009 A mutation in teg-4, which encodes a protein homologous to the SAP130 pre-mRNA splicing factor, disrupts the balance between proliferation and differentiation in the *C. elegans* germ line. Mech. Dev. 126: 417–429 19368799 10.1016/j.mod.2009.01.006

[bib35] Murata Y. Wharton R. P., 1995 Binding of pumilio to maternal hunchback mRNA is required for posterior patterning in Drosophila embryos. Cell 80: 747–756 7889568 10.1016/0092-8674(95)90353-4

[bib36] Pasierbek P. Jantsch M. Melcher M. Schleiffer A. Schweizer D., 2001 A *Caenorhabditis elegans* cohesion protein with functions in meiotic chromosome pairing and disjunction. Genes Dev. 15: 1349–1360 11390355 10.1101/gad.192701 PMC312707

[bib37] Quenault T. Lithgow T. Traven A., 2011 PUF proteins: repression, activation and mRNA localization. Trends Cell Biol. 21: 104–112 21115348 10.1016/j.tcb.2010.09.013

[bib38] Spassov D. S. Jurecic R., 2003 Mouse Pum1 and Pum2 genes, members of the Pumilio family of RNA-binding proteins, show differential expression in fetal and adult hematopoietic stem cells and progenitors. Blood Cells Mol. Dis. 30: 55–69 12667987 10.1016/s1079-9796(03)00003-2

[bib39] Stumpf C. R. Kimble J. Wickens M., 2008 A *Caenorhabditis elegans* PUF protein family with distinct RNA binding specificity. RNA 14: 1550–1557 18579869 10.1261/rna.1095908 PMC2491472

[bib40] Subramaniam K. Seydoux G., 2003 Dedifferentiation of primary spermatocytes into germ cell tumors in *C. elegans* lacking the pumilio-like protein PUF-8. Curr. Biol. 13: 134–139 12546787 10.1016/s0960-9822(03)00005-8

[bib41] Suh N. Crittenden S. L. Goldstrohm A. Hook B. Thompson B., 2009 FBF and its dual control of gld-1 expression in the *Caenorhabditis elegans* germline. Genetics 181: 1249–1260 19221201 10.1534/genetics.108.099440 PMC2666496

[bib42] Tautz D., 1988 Regulation of the Drosophila segmentation gene hunchback by two maternal morphogenetic centres. Nature 332: 281–284 2450283 10.1038/332281a0

[bib43] Varkey J. P. Jansma P. L. Minniti A. N. Ward S., 1993 The *Caenorhabditis elegans* spe-6 gene is required for major sperm protein assembly and shows second site non-complementation with an unlinked deficiency. Genetics 133: 79–86 8417991 10.1093/genetics/133.1.79 PMC1205300

[bib44] Walser C. B. Battu G. Hoier E. F. Hajnal A., 2006 Distinct roles of the Pumilio and FBF translational repressors during *C. elegans* vulval development. Development 133: 3461–3471 16908630 10.1242/dev.02496

[bib45] Wang C. Wilson-Berry L. Schedl T. Hansen D., 2012 TEG-1 CD2BP2 regulates stem cell proliferation and sex determination in the C. elegans germ line and physically interacts with the UAF-1 U2AF65 splicing factor. Dev. Dyn. 241: 505–521 22275078 10.1002/dvdy.23735 PMC3466600

[bib46] Wickens M. Bernstein D. S. Kimble J. Parker R., 2002 A PUF family portrait: 3′UTR regulation as a way of life. Trends Genet. 18: 150–157 11858839 10.1016/s0168-9525(01)02616-6

[bib47] Xu E. Y. Chang R. Salmon N. A. Reijo Pera R. A., 2007 A gene trap mutation of a murine homolog of the Drosophila stem cell factor Pumilio results in smaller testes but does not affect litter size or fertility. Mol. Reprod. Dev. 74: 912–921 17219433 10.1002/mrd.20687

[bib48] Yochem J. Greenwald I., 1989 glp-1 and lin-12, genes implicated in distinct cell-cell interactions in *C. elegans*, encode similar transmembrane proteins. Cell 58: 553–563 2758466 10.1016/0092-8674(89)90436-4

[bib49] Zetka M. C. Kawasaki I. Strome S. Muller F., 1999 Synapsis and chiasma formation in *Caenorhabditis elegans* require HIM-3, a meiotic chromosome core component that functions in chromosome segregation. Genes Dev. 13: 2258–2270 10485848 10.1101/gad.13.17.2258 PMC317003

[bib50] Zhang B. Gallegos M. Puoti A. Durkin E. Fields S., 1997 A conserved RNA-binding protein that regulates sexual fates in the *C. elegans* hermaphrodite germ line. Nature 390: 477–484 9393998 10.1038/37297

