# Supplementary material for: PUF-8, a Pumilio Homolog, Inhibits the Proliferative Fate in the Caenorhabditis elegans Germline
Source: G3 (Bethesda). 2012 Oct 1;2(10):1197–205. doi: 10.1534/g3.112.003350 (PMC3464112; doi:10.1534/g3.112.003350)
Supplement: Supporting Information [file supp_2_10_1197__index.html]

Supporting Information 

# PUF-8, a Pumilio Homolog, Inhibits the Proliferative Fate in the *Caenorhabditis elegans* Germline

## Supporting Information for Racher and Hansen, 2012

**Files in this Data Supplement:**

- Supporting Information - Figures S1 and S2 and Tables S1 and S2 (PDF, 2.4 MB)
- Figure S1 - Mapping and cloning of *teg-2(oz192)* (PDF, 371 KB)
- Figure S2 - *puf-8(oz192); glp-1(ar202)* tumor is not suppressed by *fem-3(e1996)* (PDF, 2 MB)
- Table S1 - *puf-8(q725)* enhances *glp-1(oz264)* in males at 15� (PDF, 70 KB)
- Table S2 - *puf-8(0)* does not interact with other Notch regulated cell fate decisions (PDF, 75 KB)
