# Supplementary material for: PUF-8, a Pumilio Homolog, Inhibits the Proliferative Fate in the Caenorhabditis elegans Germline
Source: G3 (Bethesda). 2012 Oct 1;2(10):1197–205. doi: 10.1534/g3.112.003350 (PMC3464112; doi:10.1534/g3.112.003350)
Supplement: Supporting Information [file supp_2.10.1197_003350SI.pdf]

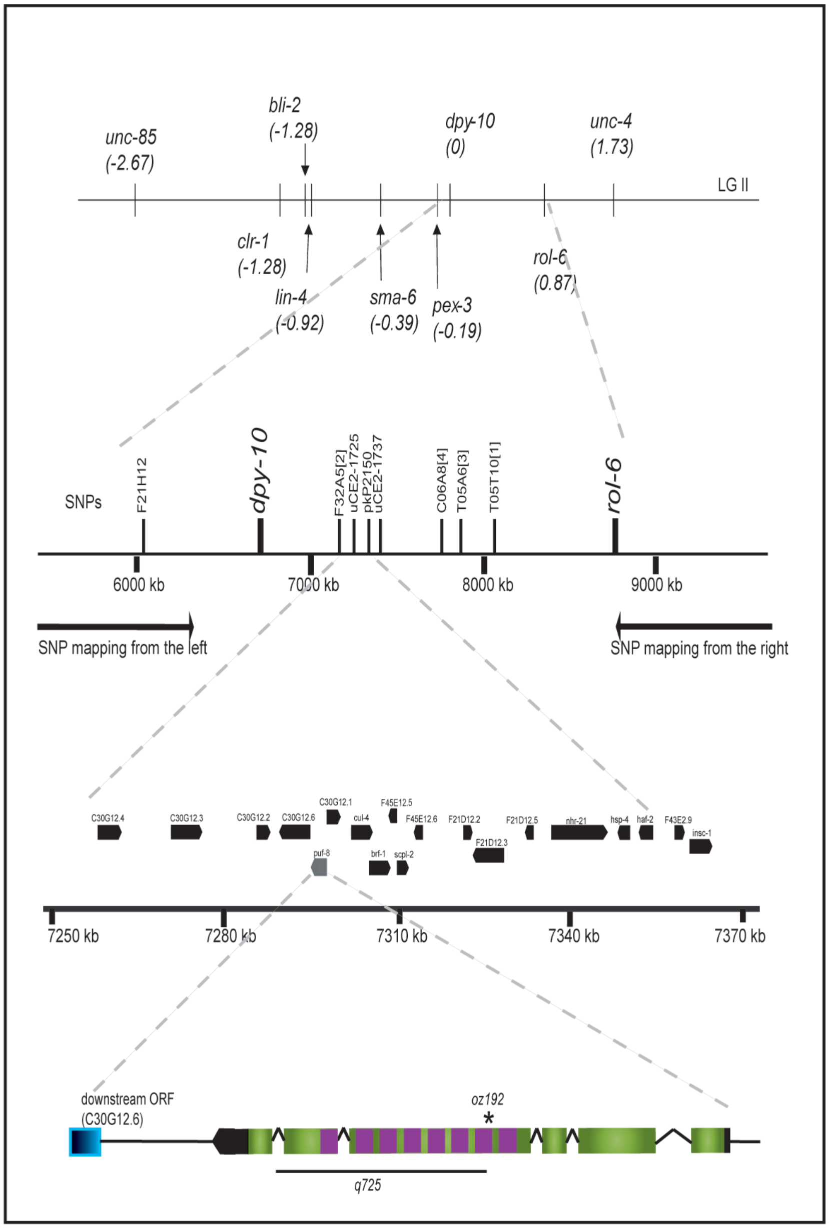

Figure S1 Mapping and cloning of *teg-2(oz192)*. We used single nucleotide polymorphism (SNP) mapping and deficiency mapping to determine that *teg-2* resides between *dpy-10* and *rol-6* on chromosome II (top map). SNP mapping was performed using the Hawaiian CB4856 *(HA-8)* strain. From *teg-2(oz192) rol-6(e187)/HA-8; glp-1(ar202gf)* and *bli-2(e768) teg-2(oz192)/HA-8; glp-1(ar202gf)* animals, we identified 24 roller non-tumorous and 34 blister non-tumorous recombinants, respectively. The furthest roller non-tumorous recombinants to the left of *rol-6(e187)* (*HA-8 rol-6(e187); glp-1(ar202gf)*) extended to SNP uCE2-1737 and the furthest blister non-tumorous recombinants to the right of *bli-2(e768)* (*bli-2(e768) HA-8; glp-1(ar202gf)*) extended to SNP F32A5[2] (second map). This narrowed the critical region containing *teg-2* to a 98kb region containing 18 genes (third map). Sequencing of one these genes, *puf-8*, revealed a G937T transversion (bottom gene model). In the gene model, black boxes represent the untranslated regions, while the green boxes represent the coding regions of the exons. The purple squares show the segments encoding the eight PUF repeats. The locations of the *puf-8(q725)* deletion and the *puf-8(oz192)* point mutation are shown.

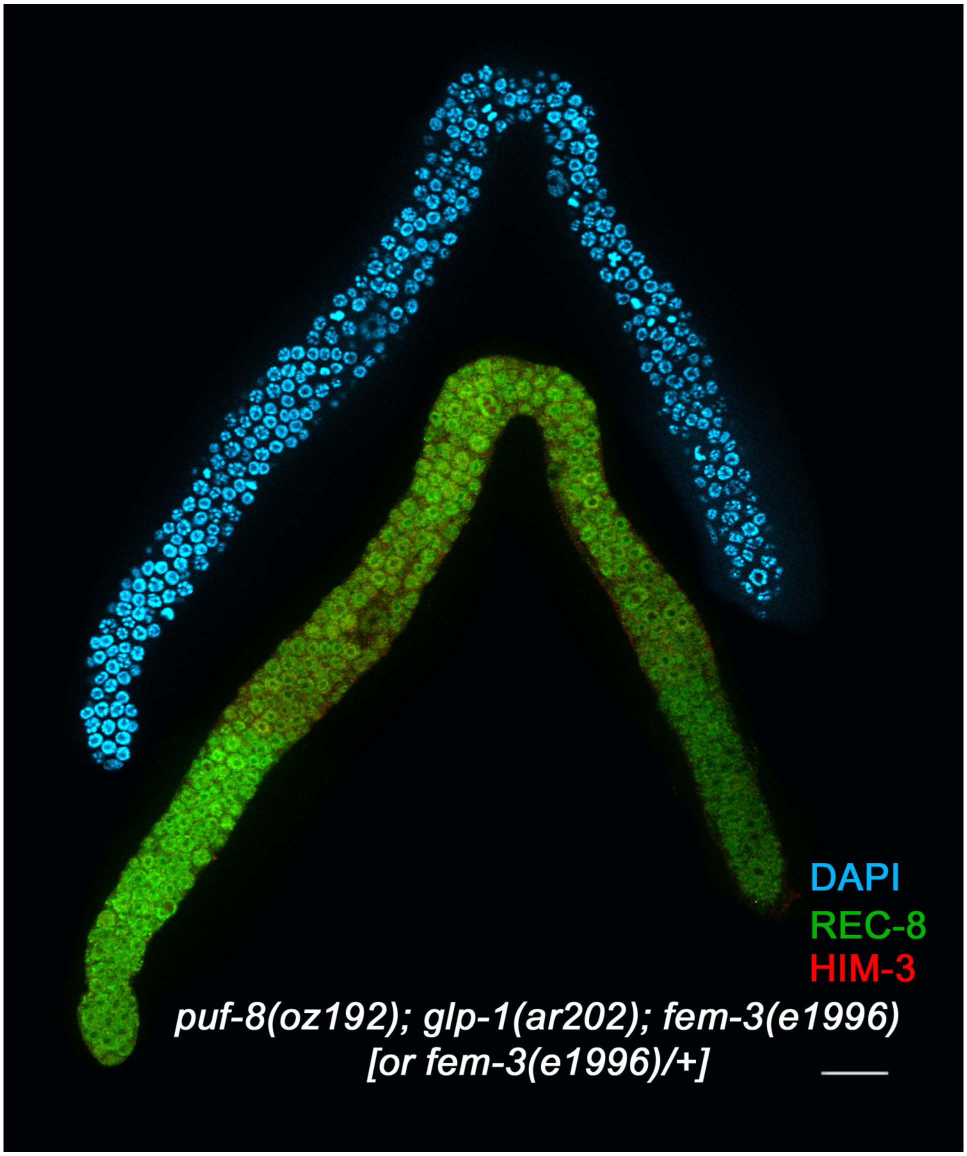

Figure S2 *puf-8(oz192); glp-1(ar202)* tumor is not suppressed by *fem-3(e1996)***.** *puf-8(oz192); glp-1(ar202)/mIn1; fem-3(e1996)/unc-24(e138) dpy-20(e1282)* animals grown at 15°C and the progeny non-green (lacking *mIn1*) and non-Unc Dpy progeny were dissected one day past the L4 stage and stained with DAPI (blue), anti-REC-8 antibodies (green) and anti-HIM-3 antibodies (red). One third of the dissected animals would be expected to be *puf-8(oz192); glp-1(ar202); fem-3(e1996)*, while two thirds would be *puf-8(oz192); glp-1(ar202); fem-3(e1996)/unc-24(e138) dpy-20(e1282)*. 33 dissected gonad arms were analyzed and all were completely tumorous (i.e. containing only anti-REC-8(+) cells, and no anti-HIM-3(+) cells).

Table S1 ***puf-8(q725)*** **enhances** ***glp-1(oz264)*** **in males at 15°**

Genotype	Tumorous[a]	n[b]
puf-8(q725)[c]	0%	50
glp-1(oz264)[d]	0%	50
puf-8(q725); glp-1(oz264)[e]	100%	54

[a] Males were scored one day past the L4 stage using DIC optics and/or using fluorescence microscopy of DAPI stained animals.
[b] All strains maintained at 15°C
[c] Maintained as a homozygous strain
[d] Maintained as a homozygous strain
[e] Obtained as cross progeny from *puf-8(q725)/mIn1; glp-1(oz264)* mothers and fathers

Table S2 ***puf-8(0)*** **does not interact with other Notch regulated cell fate decisions**

Genotype	Two Anchor Cells [a]	L1 arrest[b]	n[c]
lin-12[d]	45.7%	-	151
puf-8; lin-12[e]	43.8%	-	144
lin-12 glp-1/ unc-32[f]	-	16.1%[g]	341
puf-8; lin-12 glp-1/ unc-32[h]	-	18.1%	188

[a] In wild-type animals, two equivalent cells (Z1.ppp and Z4.aaa) interact with one another such that one cell becomes the Anchor Cell (AC), while the other becomes the Ventral Uterine precursor cell (VU). When LIN-12/Notch signalling is reduced, both cells may adopt the AC fate, depending on the degree to which LIN-12/Notch signalling is reduced; *lin-12(ar170)* is a partial loss-of-function allele, causing a portion of animals to adopt the 2AC phenotype (Hubbard *et al.* 1996). The AC was identified through the use of the *arIs51* integrated array (Karp and Greenwald 2003).

[b] Animals lacking both *glp-1* and *lin-12* activity arrest in the first larval stage (Lambie and Kimble 1991).

[c] All strains maintained at 20°C

[d] Actual genotype *unc-4(e120); unc-32(e189) lin-12(ar170); arIs51[cdh-3::gfp]*

[e] Actual genotype *puf-8(oz192) unc-4(e120); unc-32(e189) lin-12(ar170); arIs51[cdh-3::gfp]*

[f] Actual genotype *lin-12(q269) glp-1(q231)/ unc-32(e189)*

[g] We would expect 25% of animals from the *lin-12 glp-1/ unc-32* strain to segregate L1 arrested animals if *glp-1* or *lin-12* activity were completely removed; however, we only observed 16.1%. The *lin-12(q231)* is a temperature sensitive allele that only partially reduces *lin-12* activity. At 20°C, it was previously reported that 100% of *lin-12(q269) glp-1(q231)* animals are L1 arrested (Lambie and Kimble 1991); therefore, our temperature used or conditions of growth must be slightly different.

[h] Actual genotype *puf-8(q725); lin-12(q269) glp-1(q231)/ unc-32(e189)*
