# Supplementary material for: PUF-8, a Pumilio Homolog, Inhibits the Proliferative Fate in the Caenorhabditis elegans Germline
Source: G3 (Bethesda). 2012 Oct 1;2(10):1197–205. doi: 10.1534/g3.112.003350 (PMC3464112; doi:10.1534/g3.112.003350)
Supplement: Supporting Information [file supp_2.10.1197_FigureS1.pdf]

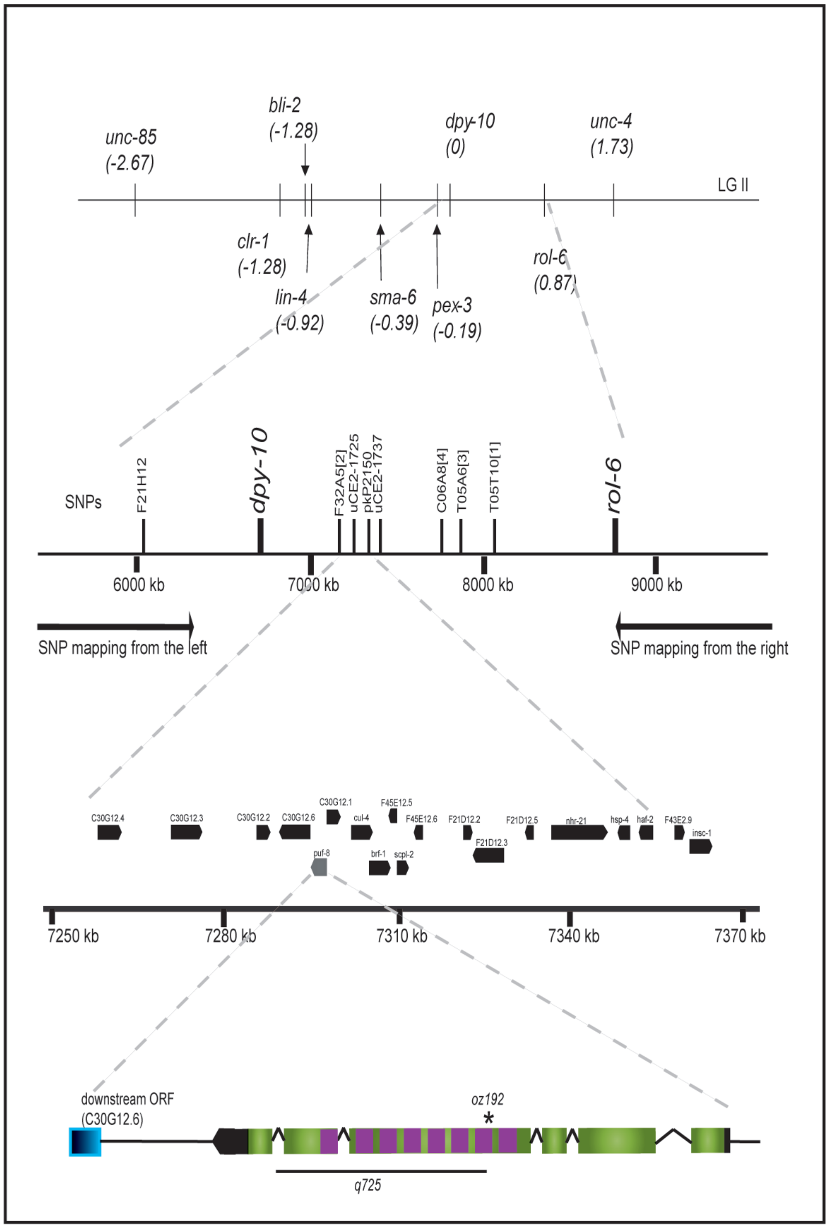

Figure S1 Mapping and cloning of *teg-2(oz192)*. We used single nucleotide polymorphism (SNP) mapping and deficiency mapping to determine that *teg-2* resides between *dpy-10* and *rol-6* on chromosome II (top map). SNP mapping was performed using the Hawaiian CB4856 *(HA-8)* strain. From *teg-2(oz192) rol-6(e187)/HA-8; glp-1(ar202gf)* and *bli-2(e768) teg-2(oz192)/HA-8; glp-1(ar202gf)* animals, we identified 24 roller non-tumorous and 34 blister non-tumorous recombinants, respectively. The furthest roller non-tumorous recombinants to the left of *rol-6(e187)* (*HA-8 rol-6(e187); glp-1(ar202gf)*) extended to SNP uCE2-1737 and the furthest blister non-tumorous recombinants to the right of *bli-2(e768)* (*bli-2(e768) HA-8; glp-1(ar202gf)*) extended to SNP F32A5[2] (second map). This narrowed the critical region containing *teg-2* to a 98kb region containing 18 genes (third map). Sequencing of one these genes, *puf-8*, revealed a G937T transversion (bottom gene model). In the gene model, black boxes represent the untranslated regions, while the green boxes represent the coding regions of the exons. The purple squares show the segments encoding the eight PUF repeats. The locations of the *puf-8(q725)* deletion and the *puf-8(oz192)* point mutation are shown.
