# Supplementary material for: PUF-8, a Pumilio Homolog, Inhibits the Proliferative Fate in the Caenorhabditis elegans Germline
Source: G3 (Bethesda). 2012 Oct 1;2(10):1197–205. doi: 10.1534/g3.112.003350 (PMC3464112; doi:10.1534/g3.112.003350)
Supplement: Supporting Information [file supp_2.10.1197_FigureS2.pdf]

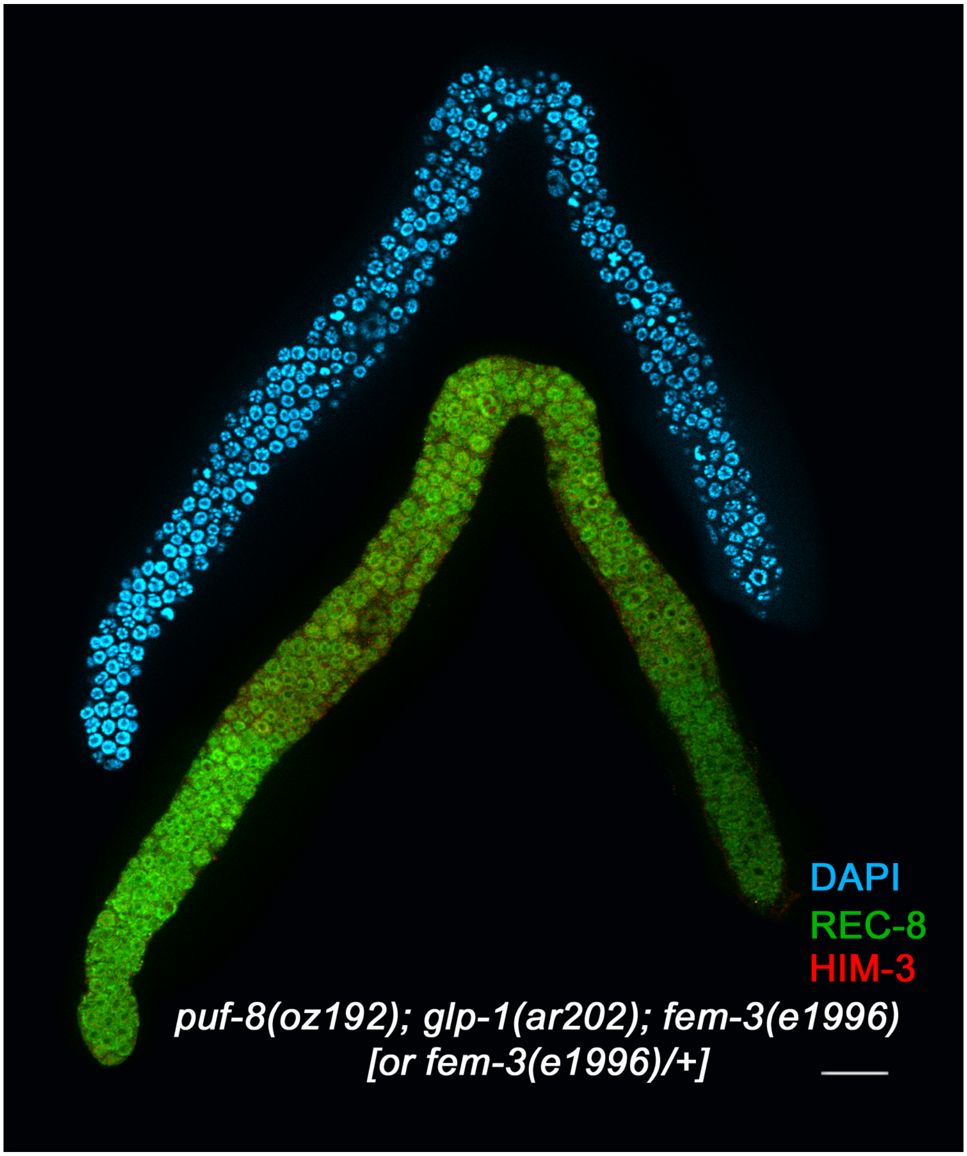

Figure S2 *puf-8(oz192); glp-1(ar202)* tumor is not suppressed by *fem-3(e1996)***.** *puf-8(oz192); glp-1(ar202)/mIn1; fem-3(e1996)/unc-24(e138) dpy-20(e1282)* animals grown at 15°C and the progeny non-green (lacking *mIn1*) and non-Unc Dpy progeny were dissected one day past the L4 stage and stained with DAPI (blue), anti-REC-8 antibodies (green) and anti-HIM-3 antibodies (red). One third of the dissected animals would be expected to be *puf-8(oz192); glp-1(ar202); fem-3(e1996)*, while two thirds would be *puf-8(oz192); glp-1(ar202); fem-3(e1996)/unc-24(e138) dpy-20(e1282)*. 33 dissected gonad arms were analyzed and all were completely tumorous (i.e. containing only anti-REC-8(+) cells, and no anti-HIM-3(+) cells).
