# Supplementary material for: PUF-8, a Pumilio Homolog, Inhibits the Proliferative Fate in the Caenorhabditis elegans Germline
Source: G3 (Bethesda). 2012 Oct 1;2(10):1197–205. doi: 10.1534/g3.112.003350 (PMC3464112; doi:10.1534/g3.112.003350)
Supplement: Supporting Information [file supp_2.10.1197_TableS1.pdf]

Table S1 *puf-8(q725)* enhances *glp-1(oz264)* in males at 15°

Genotype	Tumorous[a]	n[b]
puf-8(q725)[c]	0%	50
glp-1(oz264)[d]	0%	50
puf-8(q725); glp-1(oz264)[e]	100%	54

[a] Males were scored one day past the L4 stage using DIC optics and/or using fluorescence microscopy of DAPI stained animals.
[b] All strains maintained at 15°C
[c] Maintained as a homozygous strain
[d] Maintained as a homozygous strain
[e] Obtained as cross progeny from *puf-8(q725)/mIn1; glp-1(oz264)* mothers and fathers
