# Supplementary material for: PUF-8, a Pumilio Homolog, Inhibits the Proliferative Fate in the Caenorhabditis elegans Germline
Source: G3 (Bethesda). 2012 Oct 1;2(10):1197–205. doi: 10.1534/g3.112.003350 (PMC3464112; doi:10.1534/g3.112.003350)
Supplement: Supporting Information [file supp_2.10.1197_TableS2.pdf]

Table S2 ***puf-8(0)*** **does not interact with other Notch regulated cell fate decisions**

Genotype	Two Anchor Cells [a]	L1 arrest[b]	n[c]
lin-12[d]	45.7%	-	151
puf-8; lin-12[e]	43.8%	-	144
lin-12 glp-1/ unc-32[f]	-	16.1%[g]	341
puf-8; lin-12 glp-1/ unc-32[h]	-	18.1%	188

[a] In wild-type animals, two equivalent cells (Z1.ppp and Z4.aaa) interact with one another such that one cell becomes the Anchor Cell (AC), while the other becomes the Ventral Uterine precursor cell (VU). When LIN-12/Notch signalling is reduced, both cells may adopt the AC fate, depending on the degree to which LIN-12/Notch signalling is reduced; *lin-12(ar170)* is a partial loss-of-function allele, causing a portion of animals to adopt the 2AC phenotype (HUBBARD *et al.* 1996). The AC was identified through the use of the *arIs51* integrated array (KARP and GREENWALD 2003).

[b] Animals lacking both *glp-1* and *lin-12* activity arrest in the first larval stage (LAMBIE and KIMBLE 1991).

[c] All strains maintained at 20°C

[d] Actual genotype *unc-4(e120); unc-32(e189) lin-12(ar170); arIs51[cdh-3::gfp]*

[e] Actual genotype *puf-8(oz192) unc-4(e120); unc-32(e189) lin-12(ar170); arIs51[cdh-3::gfp]*

[f] Actual genotype *lin-12(q269) glp-1(q231)/ unc-32(e189)*

[g] We would expect 25% of animals from the *lin-12 glp-1/ unc-32* strain to segregate L1 arrested animals if *glp-1* or *lin-12* activity were completely removed; however, we only observed 16.1%. The *lin-12(q231)* is a temperature sensitive allele that only partially reduces *lin-12* activity. At 20°C, it was previously reported that 100% of *lin-12(q269) glp-1(q231)* animals are L1 arrested (LAMBIE and KIMBLE 1991); therefore, our temperature used or conditions of growth must be slightly different.

[h] Actual genotype *puf-8(q725); lin-12(q269) glp-1(q231)/ unc-32(e189)*
